# Ultrafast Exciton and Spin Dynamics of Monolayer MoSi_2_N_4_ Studied by Non‐Degenerate Pump‐Probe Transient Transmission Spectroscopy

**DOI:** 10.1002/advs.202417209

**Published:** 2025-03-07

**Authors:** Huiping Wu, Su Sun, Chuan Xu, Ke Chen, Wencai Ren, Tianshu Lai

**Affiliations:** ^1^ School of Physics State Key Lab of Optoelectronic Materials and Technologies Sun Yat‐Sen University Guangzhou Guangdong 510275 P. R. China; ^2^ Shenyang National Laboratory for Materials Science Institute of Metal Research Chinese Academy of Sciences Shenyang 110016 P. R. China; ^3^ Center for Neutron Science and Technology Guangdong Provincial Key Laboratory of Magnetoelectric Physics and Devices School of Physics Sun Yat‐sen University Guangzhou 510275 P. R. China

**Keywords:** exciton dynamics, monolayer MoSi_2_N_4_, spin dynamics, time‐resolved Faraday rotation spectroscopy, transient differential transmission spectroscopy

## Abstract

2D materials have attracted numerous attention for their potential applications in nano‐photoelectronic and valleytronic devices. Recently, a new 2D material, MoSi_2_N_4_ monolayer, is synthesized and reported, and predicted to have many unique properties. Here, its ultrafast photoelectron and spin dynamics using femtosecond‐resolved transient differential transmission and Faraday rotation spectroscopies are investigated. Complex and diverse ultrafast dynamics of excitons are observed with increasing probe wavelength from 510 to 640 nm, including fully positive and negative monotonic decaying dynamics as well as an initial positive (negative) peak followed by slow negative (positive) recovery dynamics, and are explained well based on the band structure of MoSi_2_N_4_ monolayer containing a deep defect level locating above the midpoint of direct bandgap at K valley. Spin polarization of A and B excitons is found controlled by the circular helicity of exciting light. The spin relaxation lifetime of B excitons is determined as ≈0.73 ps.

## Graphical abstract

The resonant energy of the A and B exciton of the new 2D material, monolayer MoSi_2_N_4_, is found to be 2.18 and 2.34 eV, respectively, indicating a spin‐splitting energy of ≈160 meV. Complex and diverse exciton dynamics are observed. The work has a profound influence on the future developments of MoSi_2_N_4_‐based optoelectronic devices and the synthesis of high‐quality MoSi_2_N_4_.

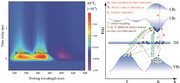

## 1 Introduction

Since graphene was first exfoliated successfully and studied in 2004,^[^ ^1^ ^]^ 2D materials have attracted increasing interest for their outstanding optoelectronic properties, such as enhanced light‐matter interactions and atomic scalability. A variety of other monolayer 2D materials have been explored and found, such as TMDCs,^[^ ^2^, ^3^ ^]^ MXenes (Ti_3_AlC_2_, Ti_2_AlC, Ta_4_AlC_3_, etc.),^[^ ^4^, ^5^ ^]^ hexagonal boron nitrides (h‐BN)^[^ ^6^ ^]^ and phosphorene^[^ ^7^ ^]^ etc. Recently, a monolayer 2D vdW material without existing 3D parents, MoSi_2_N_4_, was synthesized by the chemical vapor deposition (CVD) method.^[^ ^8^ ^]^ It consists of seven atomic layers in the order of N─Si─N─Mo─N─Si─N, which might be viewed as a MoN_2_ layer sandwiched between two Si‐N bilayers. It has excellent ambient stability, high optical transparency, high carrier mobility, and thermal conductivity.^[^ ^8^, ^9^, ^10^, ^11^, ^12^, ^13^, ^14^ ^]^ These remarkable properties make MoSi_2_N_4_ highly promising for next‐generation optoelectronic device applications such as photodetectors, light‐harvesting devices, photodiodes, and flexible optoelectronic devices.^[^ ^15^, ^16^, ^17^, ^18^, ^19^ ^]^

MoSi_2_N_4_ was theoretically predicted to possess an indirect bandgap of 1.94 eV, with the conduction band minimum (CBM) located at the K/K′ valley and the valence band maximum (VBM) at the Γ valley, which have been experimentally confirmed.^[^ ^8^ ^]^ Additionally, spin‐splitting direct‐gap A and B excitons were also predicted to exist at the K/K′ valley.^[^ ^8^, ^20^, ^21^ ^]^ The CBM of both A and B excitons are identical, while their VBM showed a splitting of 129–172 meV due to inversion symmetry breaking and strong spin‐orbit coupling,^[^ ^8^, ^20^, ^21^ ^]^ indicating great potential for MoSi_2_N_4_ in valleytronic devices.^[^ ^22^, ^23^, ^24^ ^]^ However, despite a few of theoretical calculations reported on monolayer MoSi_2_N_4_,^[^ ^25^, ^26^, ^27^ ^]^ no experimental studies on its optoelectronic responses have been reported yet. Moreover, these calculations yielded significantly disparate exciton lifetimes. For applications involving photodetectors or valleytronic optoelectronic devices, understanding the recombination lifetime and mechanism as well as a spin‐relaxation lifetime of photoexcited carriers or excitons is crucial since they directly impact the response bandwidth of such optoelectronic devices. Furthermore, recent theoretical research has shown that intrinsic point defects like N mono‐ and di‐vacancies inevitably form during synthesis and can act as recombination centers that reduce carrier lifetime;^[^ ^28^ ^]^ however, these defect states were not considered in previous lifetime calculations.^[^ ^25^, ^26^, ^27^ ^]^ Therefore, investigating the ultrafast optoelectronic response of MoSi_2_N_4_ is essential for future applications.^[^ ^25^, ^27^, ^29^, ^30^ ^]^

In this work, we investigate the ultrafast exciton and spin dynamics of monolayer MoSi_2_N_4_ grown by chemical vapor deposition (CVD) using femtosecond‐resolved pump‐probe transient transmission spectroscopy and Faraday rotation spectroscopy. The dynamics are measured under ambient conditions with variations in probe wavelength and pump fluence. We observe resonant energies of ≈2.18 and 2.34 eV for the A and B excitons, respectively, indicating a spin‐splitting energy of ≈160 meV between them. The exciton dynamics exhibit significant diversity depending on the probe wavelength, which can be attributed to the competition among three dynamic processes: formation, and recombination of two kinds of defect‐bound excitons. To accurately describe these diverse ultrafast exciton dynamics, we develop a three‐exponent model that fits well with our experimental results. By fitting the model, we obtain time constants for each dynamic process. Furthermore, we find that the spin relaxation time of the B exciton is below one picosecond.

## 2 Results and Discussion

### 2.1 Sample Characterization

**Figure 1:**
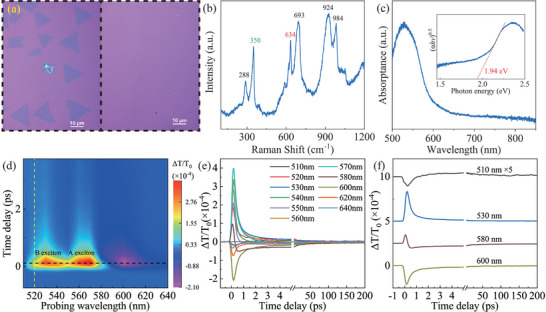
Characterization and diverse ultrafast dynamics of MoSi_2_N_4_ monolayer. a) Optical images of dispersed single crystal (left panel) and continuous poly‐crystal monolayer (right panel) transferred onto SiO_2_/Si substrate. b) Raman spectrum of MoSi_2_N_4_ monolayer on 0.5 mm thick quartz substrate with the excitation of 514 nm cw laser. c) Optical absorption spectrum of the MoSi_2_N_4_ monolayer. Inset: Tauc plot of the absorption spectrum gives out an indirect bandgap of 1.94 eV. d) Color mapping of transient differential transmission as a function of probe wavelength and delayed time. The yellow vertical dashed line at 520 nm denotes the wavelength of the pump pulse. The red stripes at around 570 and 530 nm show the position of A and B excitons, respectively. e) Diverse dynamic profiles for different probing wavelengths ranging from 510 to 640 nm. f) Typical representatives of diverse dynamics which are plotted with an offset for clarity. The photo‐injected exciton density is fixed at 0.96 × 10^12^ cm^−2^.

Dispersed single crystalline and continuous poly‐crystalline monolayer MoSi_2_N_4_ is shown in the left and right panels of **Figure** **1**a. The latter is used for all measurements in this paper. Its Raman and optical absorption spectra are shown in Figure 1b,c, respectively. Four Raman peaks at 288, 693, 924, and 984 cm^−1^ can be attributed to the vibrational modes of four different Si─N bonds,^[^ ^8^ ^]^ while two peaks at 634 and 350 cm^−1^ can be ascribed to the vibrational modes of Mo─N and Mo─Si─N bonds,^[^ ^8^ ^]^ respectively. An indirect bandgap of 1.94 eV is obtained from the fitting of the Tauc plot of the absorption spectrum (Figure 1c), and agrees well with that reported in refs. [8, 31].

We measure the probe‐wavelength dependent ultrafast dynamics of photo‐injected excitons excited by a 100 fs pump pulse at the central wavelength of 520 nm. Transient differential transmission, ΔT/T_0_, is recorded as a function of pump‐probe delay time for all probe wavelengths between 510 and 640 nm. Figure 1d presents the color mapping of ΔT/T_0_ intensity with respect to pump‐probe delay time and probe wavelength. Notably, two distinct red stripes centered around 530 nm and 570 nm are clearly observed near a delay time of 0 ps, indicating resonant energies of B and A excitons at ≈2.34 eV (530 nm) and 2.18 eV (570 nm), respectively. This energy difference between A and B excitons (≈160 meV) is consistent with the theoretically predicted spin‐splitting energy in the valence band at K/K′ valley.^[^ ^8^, ^20^ ^]^ The excitons were predominantly generated through the direct transition at the K/K′ valley, whereas indirect transitions contribute negligibly due to the significantly reduced absorption coefficients.

### 2.2 Exciton Dynamics

**Figure 2:**
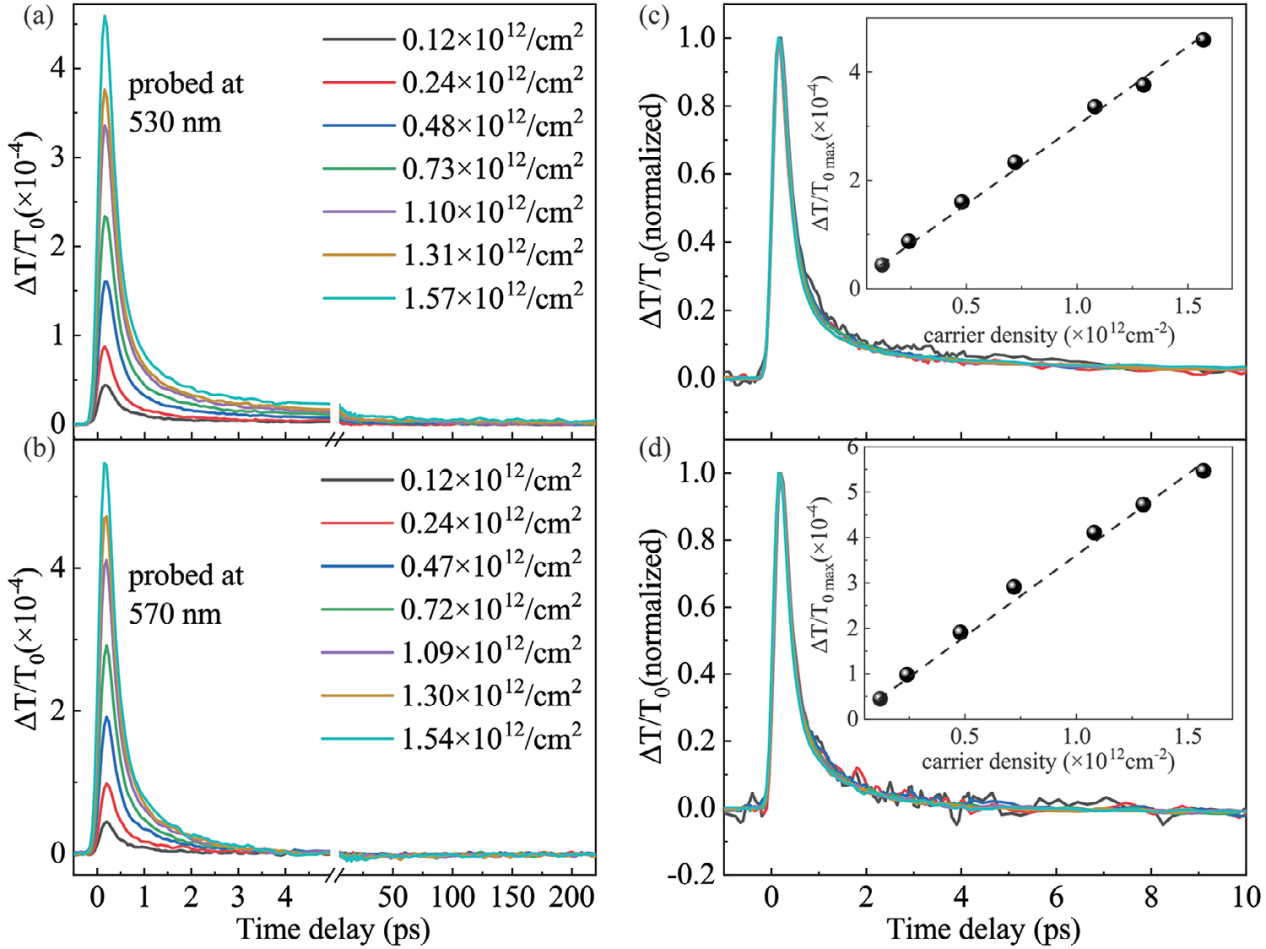
Photo‐injected exciton density‐dependent transient differential transmission dynamics (DTD) probed at 530 nm a) and 570 nm b). Panel (c,d) show the normalized DTD corresponding to (a) and (b), respectively, within the initial 10 ps. Insets: the peak amplitude in DTD as a function of the photo‐exciton density.

**Figure 3:**
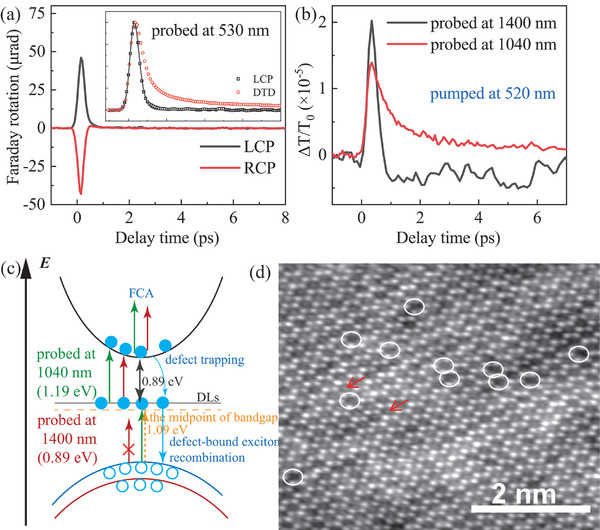
a) Spin relaxation dynamics of B exciton measured by transient Faraday rotation spectroscopy at a probe wavelength of 530 nm and under the excitation of left‐circularly‐polarized (LCP) and right‐circularly‐polarized (RCP) light at 530 nm. Inset: The normalized dynamics of Faraday rotation (black open squares) and DTD (red open circles) probed at 530 nm. The black solid line is a fitting to the dynamics of Faraday rotation. b) DTDs probed at 1040 and 1400 nm under the excitation of 520 nm with a photo‐injected exciton density of 2.41 × 10^12^ cm^−2^. c) The schematic diagram of band structures at K points of MoSi_2_N_4_ monolayer with defect levels (DLs) located above the midpoint of the direct bandgap. Possible transitions corresponding to the probe of 1040 and 1400 nm are indicated by color arrows. The filled circles signify the photo‐injected electrons, and the open circles denote the holes. FCA means free carrier absorption. d) In‐plane atomic‐level integrated differential phase contrast–STEM (iDPC‐STEM) image of monolayer MoSi_2_N_4_. The red arrows indicate the nitrogen atoms in the center of the honeycomb lattice, while the white single circles denote the nitrogen mono‐vacancies, and the white double circles display the nitrogen di‐vacancies.

To clearly understand the dynamics of excitons, multiple dynamic profiles at different probe wavelengths are presented in Figure 1e, with representative examples shown in Figure 1f for clarity. It is evident that the dynamics exhibit complex evolution and remarkable diversity. To comprehend this diversity, it is crucial to investigate the physical origin of the signal or the relaxation pathways of excitons within the optical bands. Therefore, we examine the evolution of A and B exciton dynamics with varying injected exciton densities, as depicted in **Figure** **2**a,b for B exciton and A exciton respectively. The dynamics of A (570 nm) and B (530 nm) excitons display an initial strong positive peak around zero delayed time, followed by monotonous decay toward the background through fast (<1 ps) and slow (≈100 ps) two‐component processes. Additionally, the amplitude of the initial positive peak almost increases linearly with the injected exciton densities, as illustrated in the insets in Figure 2c,d. The initial positive peak arises from the band‐filling effect.^[^ ^32^, ^33^ ^]^ Then, its rapid decaying implies the rapid decay of the exciton population in the optical bands. To comprehend the origin of exciton decay, we present the normalized density‐dependent dynamics in Figure 2c,d over a duration of 10 ps. Surprisingly, these normalized dynamics exhibit almost no dependence on the density of photo‐injected excitons. Such density‐independent decaying dynamics are characteristic of Shockley–Read–Hall (SRH) recombination,^[^ ^34^ ^]^ as opposed to free exciton annihilation^[^ ^35^, ^36^, ^37^ ^]^ or Auger recombination,^[^ ^38^ ^]^ both of which would typically display a dependence on exciton densities. Although intervalley hole scattering may also be density‐independent and lead to a rapid decay of the transient transmission, it can be ruled out based on the dynamics probed at 1400 nm in Figure 3b, as will be discussed later. Therefore, SRH recombination is determined as a unique relaxation path of free excitons. The so‐called SRH recombination is actually free excitons trapped by defect levels (DL).^[^ ^39^, ^40^ ^]^ Therefore, we can deduce that the free excitons in the optical bands are rapidly trapped into DL within the bandgap, leading to the prompt decay observed in the initial positive peaks within A and B exciton dynamics.

To further support this deduction, we employed time‐resolved Faraday rotation spectroscopy to measure the spin relaxation dynamics of B excitons, as depicted in **Figure** **3**a. The spin relaxation dynamics exhibit a prominent initial peak at zero delayed time, with the sign of the peak dependent on the helicity (LCP or RCP) of the exciting pulses. The sign changing of the Faraday rotation confirms that the initial peaks indeed originate from the spin dynamics of free excitons because LCP and RCP pump pulses can excite exciton spins with opposite orientations. Notably, unlike exciton dynamics which display weak slow recovery processes, spin relaxation dynamics exhibit that the initial peak rapidly decays to a background level close to zero. This is evident from comparing and analyzing normalized Faraday rotation and ΔT/T_0_ signals plotted in the inset of Figure 3a. The weak, slow recovery dynamics in ΔT/T_0_ are attributed to defect‐bound excitons, whose resonant energy is presumably much lower than the energy of the probe photons, thus resulting in the absence of the weak, slow recovery dynamics in Faraday rotation dynamics because the Faraday rotation of excitons is strongly influenced by the detuning between the exciton's resonant energy and the energy of the probe photons.^[^ ^41^ ^]^ Therefore, we further confirm that photo‐injected free excitons are rapidly trapped by DL to form bound excitons. However, the precise location of these defect levels within the bandgap remains unknown. Consequently, we conduct further investigations into dynamics directly associated with defect levels using a probe with photon energies of 1.19 eV (1040 nm) and 0.89 eV (1400 nm), which are lower than the direct bandgap at K/K′ valley (2.18 eV) and half of the bandgap, respectively. The results are presented in Figure 3b. The dynamics observed at 1040 nm, as indicated by the red curve, is entirely positive, while that at 1400 nm, shown by the black curve, displays an initial positive peak followed by a gradual negative recovery. It can be inferred from these observations that the deep defect levels (DL) are located at 0.1 eV above the mid‐point of the bandgap. Bound excitons are formed by trapping electrons to DL instead of holes because if DL was assumed to be located 0.1 eV below the mid‐point of the bandgap, it would have been almost fully occupied due to its 0.1 eV lower energy than the intrinsic Fermi level. Consequently, transitions from the valence band to DL would have been almost completely blocked, and as a result, the dynamics observed at 1400 nm should not exhibit a strong initial peak around zero delayed time except for subsequent negative slow recovery dynamics originating from intraband absorption of excited holes. On the other hand, if DL were located at 0.1 eV above the midpoint of the bandgap and were to trap holes from the valence band instead of electrons from the conduction band, the dynamics at 1400 nm could still show a strong initial positive peak. However, this peak would be expected to increase rather than decay rapidly as holes are trapped by DL. Based on these observations, we propose a band structure model that includes DL, as illustrated in Figure 3c, where DL is located at 0.1 eV above the midpoint of the bandgap at K/K′ valley. The origin of DL can be tentatively attributed to N mono‐ and di‐vacancy defects in the MoSi_2_N_4_ monolayer, as theoretical calculations show these N vacancies are stable under ambient conditions, and their energy levels also located above the midpoint of the direct bandgap at K/K′ valley,^[^ ^28^ ^]^ and very close to the intrinsic Fermi level. Therefore, DL can be populated partially at room temperature due to thermal excitation. Moreover, we indeed observed N mono‐ and di‐vacancy defects in the MoSi_2_N_4_ monolayer using scanning TEM, as shown in Figure 3d. Therefore, it is reasonable to attribute DL to N mono‐ and di‐vacancies.

Based on the band structure in Figure 3c, the dynamics in Figure 3b can be explained well. The 1400 nm probe can only detect the transition from DL to the conduction band (CB) (DL→CB), not the transition from the valence band (VB) to DL (VB→DL). In contrast, the 1040 nm probe can detect both transitions simultaneously. When free excitons are created by the 520 nm pump pulse, the transitions VB→DL and DL→CB can be partially blocked due to the state‐filling effect (SFE) or Pauli exclusion, leading to a reduction in probe absorption and the emergence of the initial strong positive peak in both dynamics. After free excitons are trapped to form bound excitons at DL, the conduction band becomes empty again, and DL is occupied, enhancing the DL→CB transition and reducing the VB→DL transition. The enhancement of DL→CB transition increases absorption, resulting in a negative ΔT/T_0_, while the reduction of VB→DL transition decreases absorption, leading to a positive ΔT/T_0_.

For probing at 1040 nm, the competition between the transitions VB→DL and DL→CB results in a weak positive ΔT/T_0_, causing the initial strong positive peak to decay rapidly into a weak positive tail after free excitons are rapidly trapped. However, for probing at 1400 nm, the sole transition DL→CB contributes a negative ΔT/T_0_, causing the initial strong positive peak to decay rapidly into a negative tail after free excitons are trapped rapidly. Moreover, the negative tail of 1400 nm can also exclude the possible origin of intervalley hole scattering. Because if intervalley hole scattering is the dominant mechanism, the transition of DL→CB is still blocked after the holes in K valley transferring to Γ valley, resulting a positive ΔT/T_0_ rather than a negative ΔT/T_0_. In other words, the transient transmission probed at 1400 nm should be entirely positive, similar to what we observed at 1040 nm, which is contradictory to the actual transient transmission probed at 1400 nm.

**Figure 4:**
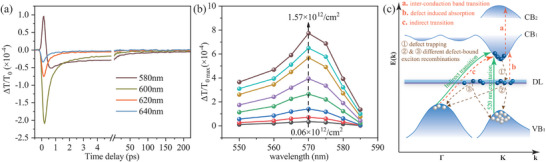
a) Typical representatives of diverse DTD probed at 580, 600, 620, and 640 nm. b) The amplitude of the peak around zero delayed time in DTD as a function of probe wavelength for different photo‐injected exciton densities. The arrow points to the increase of exciton density. c) The schematic diagram of the complex band structures of MoSi_2_N_4_ monolayer containing direct bandgap at K valley, indirect bandgap at Γ valley and defect levels. CB_1_ (VB_1_) denotes the lowest‐energy conduction band (valence band), while CB_2_ is the next lowest‐energy conduction band. DL signifies defect levels located above the midpoint of direct bandgap at K points. The green solid line arrows indicate the exciton‐injected processes via direct and indirect transitions. The brown dashed arrows labeled by ①, ②, and ③ indicate the relaxation processes, while the red dashed arrows indicate the exciton population‐induced absorption. Processes ② and ③ describe two recombination paths originating from different N mono‐ and di‐vacancy defects.

However, based on the current band structure depicted in Figure 3c, we are still unable to provide a comprehensive explanation for the fully negative dynamics observed within the 600–640 nm range, as illustrated in **Figure** **4**a. The dynamics at both 600 and 1040 nm should exhibit similar behavior since their photon energies fall below the bandgap at K/K′ valley, enabling them to simultaneously detect DL→CB and VB→DL transitions. Therefore, it remains puzzling why the dynamics probed at 600–640 nm display complete negativity while those at 1040 nm exhibit full positivity. These distinct differences strongly suggest that certain effects have been overlooked in our current model of the band structure. There are two possible effects missed, namely bandgap renormalization (BGR)^[^ ^42^ ^]^ and the transition across indirect bandgap (TIBG),^[^ ^43^ ^]^ that can be probed in the range of 600–640 nm but not at 1040 nm. To investigate the probability of the BGR effect, we conducted measurements on exciton‐density‐dependent dynamics excited at 520 nm across a wide spectrum ranging from 550 to 585 nm. Subsequently, we extracted the amplitude of the absorption saturation peak around zero delayed time. Figure 4b illustrates the plotted peak amplitude as a function of probe wavelength for various injected exciton densities. Notably, it is evident that there is no discernible redshift in the resonant absorption saturation peak of A excitons with increasing photo‐injected exciton densities, revealing negligible BGR effect. Considering that monolayer MoSi_2_N_4_ is an indirect semiconductor, the inclusion of TIBG effect in the current model of band structure (Figure 3c) is necessary. Consequently, we have incorporated TIBG into a new model of band structure, as depicted in Figure 4c. This updated model exhibits excellent agreement with previously reported theoretical calculations on the band structure,^[^ ^20^ ^]^ encompassing the transition of indirect bandgap (labeled as **c**), defect levels (labeled as DL), and inter‐conduction band transitions (labeled as **a**).^[^ ^44^, ^45^ ^]^

Based on the updated model, we can provide a comprehensive explanation for the diverse ultrafast dynamics, as depicted in Figure 1e. A pump pulse at 520 nm can simultaneously excite electrons from the valence bands at Γ (indirect transition labeled by a slanted solid arrow) and K (direct transition by a vertical solid arrow) valleys into the conduction band CB_1_, resulting in the generation of free excitons. Subsequently, within 1 ps, these free excitons are rapidly transferred to DL of N and N_2_ vacancies^[^ ^28^ ^]^ (dashed arrow labeled by ①), forming defect‐bound excitons. These bound excitons then undergo recombination with holes at K and Γ valleys, as shown by the dashed arrows labeled by ② and ③. It should be noted that processes ② and ③ are attributed to different defect‐bound recombination. It is worth emphasizing that processes ② and ③ may exhibit different recombination rates due to different types of defect levels. The diverse ultrafast dynamics observed reflect the population evolution of photo‐excited excitons in CB_1_ through three transitions of ①, ②, and ③. The dynamics probed at 510 nm exhibit an initial negative peak, followed by a rapid decay into a weak positive slow recovery. The initial negative peak arises from the dominance of re‐absorption induced by the transition process from CB_1_ to CB_2_,^[^ ^44^, ^45^ ^]^ rather than the band‐filling effect. This is supported by accurate computation of the band structure of monolayer MoSi_2_N_4_,^[^ ^20^ ^]^ which revealed that the probe at 510 nm can excite electrons in CB_1_ to CB_2_, resulting in additional stronger absorption of the probe. Subsequently, process ① occurs rapidly, leading to complete trapping of electrons to DL and thus eliminating process **a**. However, holes at K valley still contribute to the absorption saturation of the probe. Consequently, the initial negative peak rapidly decays into a positive signal due to the rapid trapping of DL. Subsequently, this positive signal gradually diminishes as recombination processes ② and ③ progress. In the spectral range of 520–570 nm, the ultrafast dynamics exhibit remarkable similarity, displaying an initial intense positive peak at zero delay time followed by a monotonic decay toward the background through two distinct dynamic processes characterized by fast and slow dynamics. The origin of the initial strong positive peak can be attributed to the band‐filling effect caused by free A and B excitons. Its rapid decay reflects the swift progression of process ①, while subsequent recovery occurs in two steps with varying rates, indicating the advancement of recombination processes ② and ③. The dynamics probed at 580 nm exhibit an initial positive peak, followed by a rapid decay to a stronger negative peak and a subsequent slow recovery. The initial positive peak still arises from the saturation absorption induced by the free exciton population. Although the photon energy of 580 nm photons is lower than the resonant energy of A exciton, the higher energy edge of the broad spectrum of femtosecond pulses with a central wavelength of 580 nm can still detect the lower‐energy exciton population at the edge of the band, resulting in a positive peak with a weaker amplitude compared to that probed at resonant energy of A excitons. The rapid decay of the initial peak reflects the rapid trapping of free excitons by DL. The subsequent generation of a stronger negative peak is attributed to nearly resonant indirect transition absorption or process **c**. Indirect transition‐induced absorption enhancement or negative ΔT/T_0_ signal has been extensively reported in materials such as Si^[^ ^46^ ^]^ and few‐layer MoS_2_.^[^ ^32^ ^]^ When probing in the wavelength range of 600–640 nm, direct detection of A and B‐free excitons is not possible, resulting in the disappearance of absorption saturation. However, the indirect transition (process **c**) can still occur, leading to a strong initial negative peak. The rapid decay of this peak reflects the fast progression of process ①, while the subsequent slow recovery is dominated by recombination processes ② and ③. Thus far, we have provided a comprehensive explanation for diverse ultrafast dynamics probed across a wide spectrum ranging from 510 to 640 nm.

### 2.3 Analysis of the Exciton Dynamics

To understand those diverse dynamics deeply, it is very necessary to analyze them quantitatively. Based on the discussions above and the model of the band structure in Figure 4c, photo‐injected excitons undergo the three dynamic processes of ①, ②, and ③. Therefore, the whole dynamic process can be well described by a tri‐exponent function. Taking account of the effect of the duration of pump and probe pulses, the practical model can be the convolution of the tri‐exponent function with a cross‐correlation function of pump and probe pulses. It can be written as

(1) $$\begin{array}{ccc} \frac{\Delta T}{T_{0}} & = & C\left(t\right)⊗\left\{\Theta \left(t\right)\cdot \left[A_{1}\exp\left(-\frac{t}{\tau_{1}}\right)+\left(1-\exp\left(-\frac{t}{\tau_{1}}\right)\right)\right.\right.\\ & & \left.\left.\left(A_{2}\exp\left(-\frac{t}{\tau_{2}}\right)+A_{3}\exp\left(-\frac{t}{\tau_{3}}\right)\right)\right]\right\} \end{array}$$

where the first exponential term describes the progress of process ①. The term (1‐exp(‐t/τ_1_)) describes the accumulation of bound excitons in DL or the building up of defect‐bound excitons, while the two exponential functions containing τ_2_ and τ_3_ do the recombination via ② and ③, respectively. *C*(*t*) is the cross‐correlation function of pump and probe pulses, and is usually described by a Gaussian function, and Θ(*t*) is a step function. A_1_ (τ_1_), A_2_ (τ_2_) and A_3_ (τ_3_) are the amplitudes (time constants) of three dynamic processes of ①, ②, and ③, respectively.

**Figure 5:**
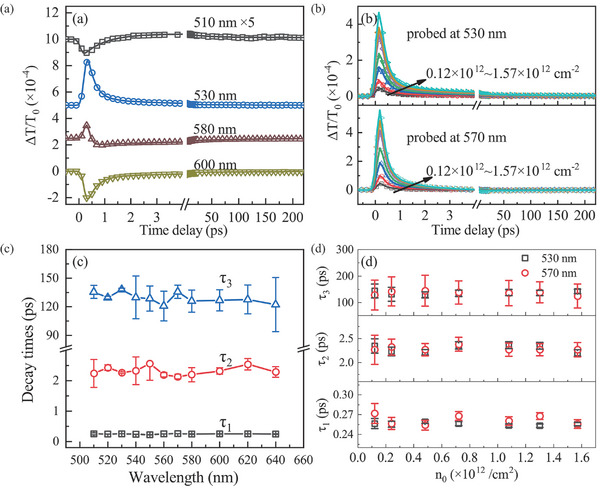
a) Fittings to four typical representatives in diverse DTD in Figure 1f. The open symbols denote experimental data and the solid lines are best fittings to experimental data with Equation (1). b) Fittings to exciton density‐dependent DTD probed at 530 and 570 nm. c) Fitting‐retrieved three‐time constants, τ_1_, τ_2_ and τ_3_, as a function of probing wavelength. d) Three time constants extracted by fitting DTD probed at 530 and 570 nm as a function of exciton density.

Equation (1) is used to fit diverse ultrafast dynamics shown in Figure 1e and Figure 2a,b. Their partial typical representatives are plotted in **Figure** **5**a, while the whole fittings are shown in Figure S1 (Supporting Information). Fittings to photo‐injected exciton density‐dependent dynamics of A and B excitons are shown in Figure 5b. The fittings agree very well with the experimental curves. The extracted lifetimes (τ_1_, τ_2_, τ_3_) of the three dynamic processes ①, ② and ③ are plotted in Figure 5c,d as a function of probe wavelength and exciton density, respectively. The amplitudes (A_1_, A_2_, A_3_) are plotted in Figure S2 (Supporting Information), and show a pronounced dependence on the probing wavelength, indicating the probe wavelength‐dependent responsivity of the three processes. It is worth noting that all lifetimes look almost independent of the probe wavelength, which is just what we expect because relaxation processes of 520 nm photo‐excited excitons are objective and only controlled by band structure and intrinsic properties of samples, and are independent of probe wavelength. Furthermore, the density‐independent time constants of τ_1_ further confirms the SRH recombination mechanism of photo‐injected excitons quantitatively. One can see from Figure 5c,d that the lifetime of excitons trapped by DL is τ_1_ ≈ 0.25 ps, revealing photo‐excitons in CB_1_ only stay ≈0.25 ps average. Then, bound excitons in DL undergo fast and slow recombination processes with time constants of τ_2_ ≈ 2.2 ps and τ_3_ ≈ 120 ps, respectively. The fast and slow recombination processes can be ascribed to different recombination rates of two types of DLs from N mono‐ and di‐vacancies. Implied by the iDPC‐STEM in Figure 3d, N mono‐vacancy has a higher density than di‐vacancies. Thus mono‐vacancy may lead to faster recombination. Therefore, we can tentatively ascribe the fast and slow recombinations to N mono‐and di‐vacancies, respectively.

We also fit the dynamics of Faraday rotation in Figure 3a with Equation (1) under setting A_2_ = A_3_ = 0, as the solid line shows in the inset. The fitting gives an effective spin lifetime, $T_{\mathrm{spin}}^{*}$ = τ_1_ = 0.15 ps. It was well‐known that, 1/$T_{\mathrm{spin}}^{*}$ = 2/T_spin_ + 1/τ_r_,^[^ ^47^ ^]^ where T_spin_ and τ_r_ is the intrinsic spin relaxation lifetime and recombination lifetime of free exciton, respectively. From Figure 5c, τ_r_ = τ_1_ = 0.25 ps for the probe wavelength of 530 nm is obtained. Therefore, the spin lifetime of photo‐injected excitons in CB_1_ can be calculated as T_spin_ = 0.73 ps. The short spin lifetime may correspond to the intrinsic spin relaxation within K or K´ valleys, and longer inter‐valley spin relaxation between K and K´ valleys cannot be observed due to the limit of the short recombination lifetime of free excitons. Such a short intravalley spin lifetime is possible due to a strong spin‐orbit coupling, also reported in MoS_2_ monolayer.^[^ ^48^ ^]^

## 3 Conclusion 

In summary, we have studied probe‐wavelength and excited‐exciton‐density dependences of ultrafast dynamics of photo‐injected exciton in monolayer MoSi_2_N_4_ under the excitation of 520 nm pulses using transient differential transmission spectroscopy. The resonant energies of A and B excitons are identified ≈2.18 and 2.34 eV, respectively, giving out a valence‐band spin‐splitting energy of 160 meV caused by the strong spin‐orbit coupling. The excited density dependence of the ultrafast dynamic of free A and B excitons reveals that their recombination is dominated by the SRH mechanism, leading to defect‐bound excitons formed in ≈0.25 ps. Then, the defect‐bound excitons recombine through fast and slow recombination processes with time constants of ≈2.2 and ≈120 ps, respectively. The defect levels are determined to be located at 0.1 eV above the midpoint of direct bandgap at K/K′ valleys. The spin relaxation dynamics of B excitons have been also studied by the time‐resolved Faraday rotation. Intravalley spin relaxation lifetime is obtained to be ≈0.73 ps. Our results provide the first important insights into the fundamental ultrafast dynamics of MoSi_2_N_4_, which may have a profound influence on its future developments of MoSi_2_N_4_‐based optoelectronic devices and the synthesis of high‐quality MoSi_2_N_4_.

## 4 Experimenta Section

### Sample Preparation and Characterization

Centimeter‐scale monolayer MoSi_2_N_4_ is grown by CVD on a Cu/Mo bilayer substrate with NH_3_ gas as the nitrogen source and a pure Si plate placed above the Cu/Mo substrate as Si source. The sample was then transferred to a 0.5 mm‐thick quartz substrate for various optical measurements. The details of the synthesis process of the sample and the characterizations are described in detail in ref. [8]

### Transient Differential Transmission Spectroscopy

A Yb‐doped femtosecond oscillator (Coherent, Chameleon Discovery) generated the pulse train of 1040 nm with a duration of 100 fs, an average power of 4.7 W, and a repetition rate of 80 MHz. It pumps an OPO oscillator. The OPO outputs a tunable wavelength from 660 to 1320 nm at port A and a fixed 1040 nm at port B. The output at port B was doubled to 520 nm by a BBO crystal to serve as the pump pulse. The output at port A was doubled to 510–640 nm by a frequency converter (A.P.E, HarmoniXX) to serve as a tunable probe. The two beams of lights from ports A and B were directed into a standard non‐collinear pump‐probe setup, and then output two beams of parallel lights which are focused on the sample by an off‐axis parabolic (OAP) mirror. The pulses of 1400 nm were generated by another optical parametric oscillator (A.P.E, Levante IR fs) under the pumping of 1040 nm femtosecond pulses. The probe light transmitted through the sample was detected with a Si photodiode (Thorlabs, PDA100A2) for the wavelength from 510 to 1040 nm and an InGaAs photodiode (Micro photons, C3A‐2300LAE) for 1400 nm. A mechanical optical chopper modulated pump pulses at a frequency of 1.333 kHz and synchronized a DSP lock‐in amplifier (SSI, OE2042). Pump‐induced differential transmission change was measured by a lock‐in amplifier as a function of delaying time of the probe pulse. All of the measurements are carried out at room temperature.

### Time‐Resolved Faraday Rotation Spectroscopy

Femtosecond time‐resolved Faraday rotation measurements are carried out with a similar experimental system to the transient differential transmission setup, but an achromatic quarter‐wave plate and polarizer are inserted in pump and probe paths before the sample, respectively. The wave plate controls the circular polarization helicity of pump pulses, while the polarizer makes the probe pulse have a higher purity of linear polarization. The probe pulses that are transmitted through the sample pass through a half‐wave plate, and then are split into two cross‐polarized pulses by a Wollaston prism. The two split probe pulses are detected by an optical balance bridge detector. Rotating the half‐wave plate can adjust the output of the balance detector to zero without pump pulses. Then, the change of the output of the balance detector just reflects the rotation angle of the probe's polarization plane induced by circular polarization pump pulse‐excited spin excitons in K or K´ valley, which is so‐called Faraday rotation because the rotation angle originates from pump‐injected exciton spin via Faraday effect. Left‐ and right‐handed circular polarization pump pulses can excite opposite spin orientations which leads to opposite Faraday rotation angles.

### In‐Plane Atomic‐Level Integrated Differential Phase Contrast–STEM (iDPC‐STEM)

The integrated differential phase contrast (iDPC) scanning transmission electron microscopy (STEM) imaging was performed on an FEI Titan Cube Themis G2 300 instrument equipped with a high‐brightness field‐emission gun (X‐FEG), double spherical aberration corrector, and a monochromator. Detailed iDPC‐STEM imaging parameters were given as follows: typical camera length (285 mm), collection semi‐angle (5–27 mrad), beam current (10 pA), and acceleration voltage (300 kV).

## Conflict of Interest

The authors declare no conflict of interest.

## Author Contributions

K.C., W.R., and T.L. supervised the project. T.L., K.C., and H.W. designed the experiments. H.W. performed the Raman spectroscopy, optical absorption spectroscopy, and all of the time‐resolved measurements with the assistance of T.L. and K.C., W.R., S.S., and C.X. provided the monolayer MoSi_2_N_4_, offered its optical images and the iDPC‐STEM image. T.L. and H.W. analyzed and interpreted the data. T.L., K.C., and H.W. wrote the paper with input from all the authors.

## Supporting information



Supporting information

## Data Availability

The data that support the findings of this study are available from the corresponding author upon reasonable request.
