# Supplementary material for: Ultrafast Exciton and Spin Dynamics of Monolayer MoSi2N4 Studied by Non‐Degenerate Pump‐Probe Transient Transmission Spectroscopy
Source: Adv Sci (Weinh). 2025 Mar 7;12(17):2417209. doi: 10.1002/advs.202417209 (PMC12061321; doi:10.1002/advs.202417209)
Supplement: Supplementary file 1 — Supporting information [file ADVS-12-2417209-s001.docx]

Supporting Information

Ultrafast Exciton Dynamics of Monolayer MoSi_2_N_4_ by Non-Degenerate Transient Transmission Spectroscopy

*Huiping Wu, Su Sun, Chuan Xu, Ke Chen*, Wencai Ren*, Tianshu Lai**


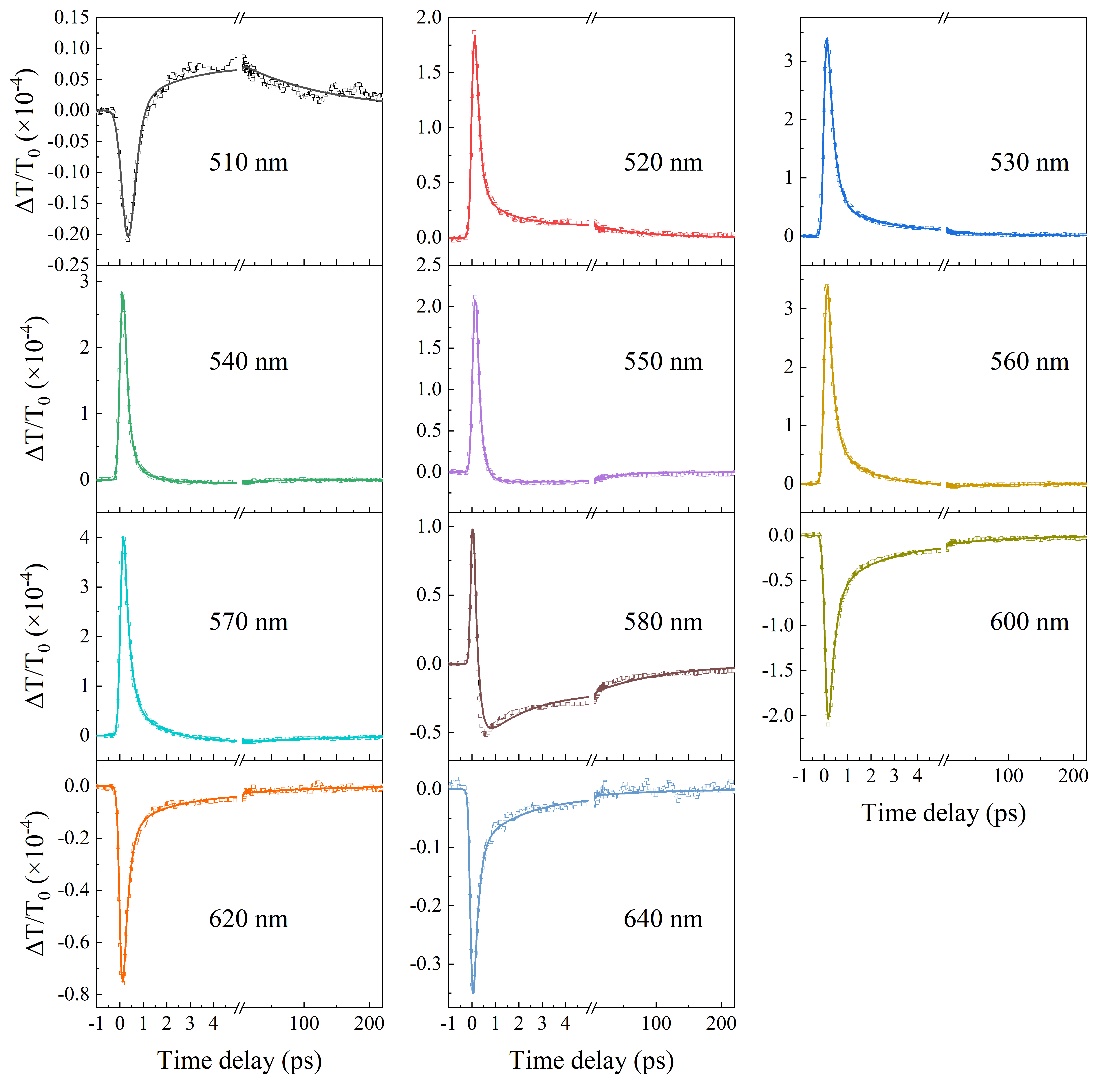


**Figure S1. Transient differential transmission dynamics probed at different wavelength.** The pump pulses is at the central wavelength of 520 nm. The curves indicated by hollow circles represent the experiment data, while the solid curves are the fittings with equation (1) in the main text.


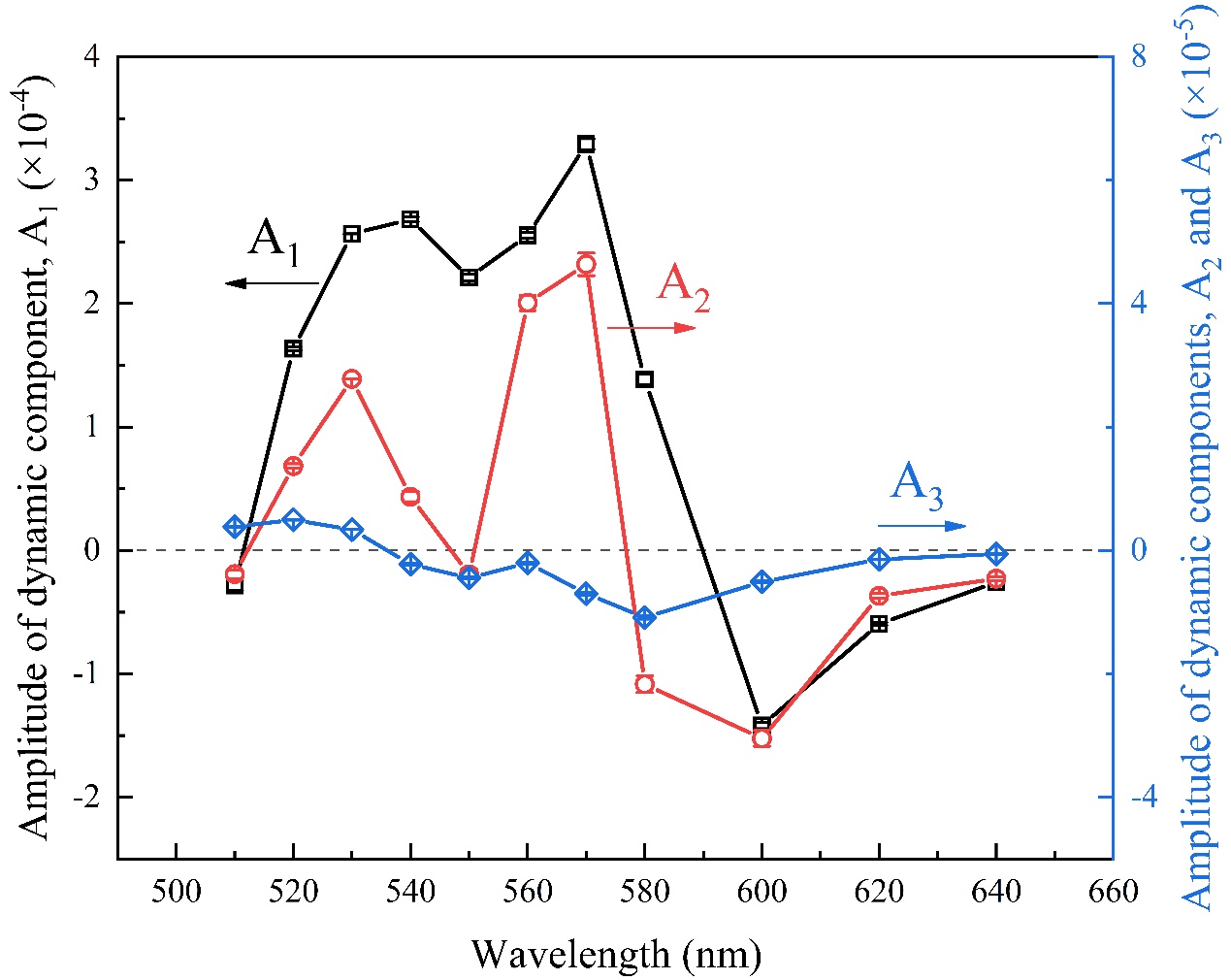


**Figure S2.** **The tri-exponential fitting amplitudes of dynamic components of the wavelength-dependent transmission spectroscopy.**

First of all, it should note that A_2_ is one order of magnitue weaker than A_1_. It is scaled by the right axis, which shows that band filling of free excitons (the e-h pairs) is much stronger than the holes alone. For the wavelength range less than 580 nm, A_1_ and A_2_ present similar positive double peak shapes with peak positions near the resonant absorption wavelengths of free A and B excitons, respeectively. Meanwhile, a minimum point between the double peaks occurs at 550 nm which is the mid-point of the resonant absorption peaks of A and B excitons. The prominent peak features of A_1_ reflect the resonant band-filling effects of the free excitons A and B, while the peak features of A_2_ correspond exclusively to the effects of the spin-split holes in the K/K' valleys after electrons have been completely trapped into the deep levels (DLs). The double-peak profiles of A_1_ and A_2_ further substantiate our inference that it is electrons, rather than holes, that are being trapped into the deep levels (DLs). This is because the energy levels of the electrons in both excitons are degenerate, which should not result in such pronounced double resonant peaks for A_1_ and A_2_. For the wavelength range above 580 nm, both A_1_ and A_2_ exhibit fully negative values, which reflect an enhanced absorption due to the indirect interband transitions between the K/K' and Γ valleys.
